# Renal Function and Risk of Coronary Heart Disease in General Populations: New Prospective Study and Systematic Review

**DOI:** 10.1371/journal.pmed.0040270

**Published:** 2007-09-04

**Authors:** Emanuele Di Angelantonio, John Danesh, Gudny Eiriksdottir, Vilmundur Gudnason

**Affiliations:** 1 Department of Public Health and Primary Care, University of Cambridge, Cambridge, United Kingdom; 2 Icelandic Heart Association, Kopavogur, Iceland; 3 University of Iceland, Reykjavik, Iceland; The George Institute, Australia

## Abstract

**Background:**

End-stage chronic kidney disease is associated with striking excesses of cardiovascular mortality, but it is uncertain to what extent renal function is related to risk of subsequent coronary heart disease (CHD) in apparently healthy adults. This study aims to quantify the association of markers of renal function with CHD risk in essentially general populations.

**Methods and Findings:**

Estimated glomerular filtration rate (eGFR) was calculated using standard prediction equations based on serum creatinine measurements made in 2,007 patients diagnosed with nonfatal myocardial infarction or coronary death during follow-up and in 3,869 people without CHD in the Reykjavik population-based cohort of 18,569 individuals. There were small and nonsignificant odds ratios (ORs) for CHD risk over most of the range in eGFR, except in the lowest category of the lowest fifth (corresponding to values of <60 ml/min/1.73m^2^), in which the OR was 1.33 (95% confidence interval 1.01–1.75) after adjustment for several established cardiovascular risk factors. Findings from the Reykjavik study were reinforced by a meta-analysis of six previous reports (identified in electronic and other databases) involving a total of 4,720 incident CHD cases (including Reykjavik), which yielded a combined risk ratio of 1.41 (95% confidence interval 1.19–1.68) in individuals with baseline eGFR less than 60 ml/min/1.73m^2^ compared with those with higher values.

**Conclusions:**

Although there are no strong associations between lower-than-average eGFR and CHD risk in apparently healthy adults over most of the range in renal function, there may be a moderate increase in CHD risk associated with very low eGFR (i.e., renal dysfunction) in the general population. These findings could have implications for the further understanding of CHD and targeting cardioprotective interventions.

## Abstract 2

John Danesh and colleagues conclude there may be a moderate increase in risk of coronary heart disease associated with very low estimated glomerular filtration rate.

## Editors' Summary

**Background.:**

Coronary heart disease (CHD), the leading cause of death in most Western countries, is a “cardiovascular” disease—literally a disorder affecting the heart and/or blood vessels. In CHD, the blood vessels that supply the heart become increasingly narrow. Eventually, the flow of blood to the heart slows or stops, causing chest pains (angina), breathlessness, and heart attacks. Many factors increase the risk of developing CHD and other cardiovascular diseases, including high blood pressure, high blood levels of cholesterol (a type of fat), or being overweight. Individuals can reduce their chances of developing cardiovascular disease by taking drugs to reduce their blood pressure or cholesterol levels or by making lifestyle changes (so-called cardioprotective interventions). Another important risk factor for cardiovascular disease is end-stage chronic kidney disease (CKD), a condition in which the kidneys stop working. (In healthy people, the kidneys remove waste products and excess fluid from the body.) People with end-stage CKD (which is treated by dialysis) have about a five times higher risk of dying from cardiovascular disease compared with healthy people.

**Why Was This Study Done?:**

End-stage CKD is preceded by a gradual loss of kidney function. There is a clear association between non-dialysis–dependent CKD and the incidence of cardiovascular events (such as heart attacks) in people who already have signs of cardiovascular disease. But are people with slightly dysfunctional kidneys (often because of increasing age) but without any obvious cardiovascular disease at greater risk of developing cardiovascular diseases than people with fully functional kidneys? If the answer is yes, it might be possible to reduce CHD deaths by minimizing the exposure of people with CKD to other risk factors for cardiovascular disease. In this study, the researchers have taken two approaches to answer this question. In a population-based study, they have examined whether there is any association in healthy adults between kidney function measured at the start of the study and incident CHD (the first occurrence of CHD) over subsequent years. In addition, they have systematically searched the published literature for similar studies and combined the results of these studies using statistical methods, a so-called “meta-analysis.”

**What Did the Researchers Do and Find?:**

Between 1967 and 1991, nearly 19,000 middle-aged men and women without a history of heart attacks living in Reykjavik, Iceland, enrolled in a prospective study of cardiovascular disease. Baseline blood samples were taken at enrollment and the participants' health monitored for 20 years on average. The researchers identified 2,007 participants who suffered a nonfatal heart attack or died of CHD during follow-up and 3,869 who remained disease free. They then calculated the estimated glomerular filtration rate (eGFR; a measure of kidney function) for each participant from baseline creatinine measurements (creatinine is a muscle waste product). There was no association between lower-than-average eGFRs and the risk of developing CHD over most of the range of eGFR values. However, people whose eGFR was below approximately 60 units had about a 40% higher risk of developing CHD after allowing for established cardiovascular risk factors than individuals with higher eGFRs. This finding was confirmed by the meta-analysis of six previous studies, which included a further 2,700 incident CHD cases.

**What Do These Findings Mean?:**

These findings indicate that people with an eGFR below about 60 units (the cut-off used to define CKD) may have an increased risk of developing CHD. They also indicate a nonliner association between kidney function and CHD risk. That is, any association with CHD became evident only when the eGFR dropped below about 60 units. These findings need confirming in different ethnic groups and by using more accurate methods to measure eGFRs. Nevertheless, they suggest that improving kidney function across the board is unlikely to have much effect on the overall incidence of CHD. Instead, they suggest that targeting cardioprotective interventions at the one in ten adults in Western countries whose eGFR is below 60 units might be a good way to reduce the burden of CHD.

**Additional Information.:**

Please access these Web sites via the online version of this summary at http://dx.doi.org/10.1371/journal.pmed.0040270.

- MedlinePlus encyclopedia pages on coronary heart disease, chronic kidney failure, and end-stage kidney disease (in English and Spanish).
- Information for patients and carers from the American Heart Association on all aspects of heart disease, including prevention of CHD
- Information from the British Heart Foundation on heart disease and on keeping the heart healthy
- Information on chronic kidney disease from the US National Kidney Foundation, and the US National Kidney and Urologic Diseases Information Clearing House (in English and Spanish)
- Information on chronic kidney disease from the UK National Kidney Foundation

## Introduction

There is a striking excess (i.e., relative risks of ≈5) of cardiovascular disease mortality in patients with end-stage chronic kidney disease (CKD) compared with the general population [1,2]. Strong associations have also been reported between the occurrence of non-dialysis-dependent CKD and subsequent incidence of cardiovascular outcomes in high-risk groups, including patients with pre-existing ischaemic cardiovascular diseases, heart failure, and high blood pressure [3–7]. Major scientific societies have recommended that all patients with manifest cardiovascular disease should be screened for evidence of kidney disease [7]. There is, by contrast, comparatively sparse and uncertain evidence on renal dysfunction (evaluated using estimated glomerular filtration rate [eGFR]) and risk of cardiovascular diseases in people without known cardiovascular or renal diseases, even though demonstration of even modest risks in the general population could have considerable public health and clinical relevance. A recent systematic review identified only a few prospective studies of non-dialysis-dependent CKD in general populations, three of which were listed as reporting on cardiovascular outcomes, comprising a total of only about 900 cardiovascular deaths [8]. Reports from these [9–11] (and other more recently published [12–18]) studies have yielded apparently conflicting findings, and their interpretation has been further complicated by mixing of vascular outcomes related to different arterial beds (such as diseases of the coronary, cerebrovascular, and peripheral circulations).

We report new data on eGFR from the prospective population-based Reykjavik Study of almost 19,000 middle-aged Icelandic men and women monitored for a mean duration of about 20 years [19]. After exclusion of participants with evidence of baseline cardiovascular diseases or diabetes, nested case-control analyses involved 2,007 incident cases with either first-ever nonfatal myocardial infarction (MI) or coronary death and 3,869 controls, almost twice as many CHD cases as in the previous largest population-based report [16]. We investigated associations with a wide range of established and emerging markers (such as those related to lipid, inflammatory, fibrinolytic, and metabolic pathways) in order to better understand the correlates (and possible determinants) of eGFR. We contextualized our new data by conducting an updated meta-analysis of published reports on renal function and incident CHD, involving a further 2,713 CHD cases from nine population-based prospective studies (identified in six previous reports).

## Methods

### Participants

The Reykjavik Study, initiated in 1967 as a prospective study of cardiovascular disease, has been described in detail previously [19]. Briefly, all men born between 1907 and 1934 and all women born between 1908 and 1935 who were residents of Reykjavik, Iceland, and its adjacent communities on 01 December 1966, were identified in the national population register and then invited to participate in the study during five stages of recruitment between 1967 and 1991. A total of 8,888 men and 9,681 women without a history of myocardial infarction were enrolled, reflecting a response rate of 72% percent. Nurses administered questionnaires, made physical measurements, recorded an electrocardiogram, performed spirometry, and collected fasting venous blood samples for preparation of aliquots of serum, which were stored at −20 °C for subsequent analysis. All participants have been monitored subsequently for cause-specific mortality and for cardiovascular morbidity, with a loss to follow-up of only about 0.6% to date. A total of 2,007 men and women with available serum creatinine samples and without cardiovascular diseases or diabetes at baseline (i.e., participants with self-reported diabetes or a fasting blood glucose of >7 mmol/l were excluded) had major coronary events between the beginning of follow-up and the censoring date. Deaths from coronary heart disease were ascertained from central registers on the basis of a death certificate with International Classification of Diseases version 9 (ICD-9) codes 410–414, and the diagnosis of nonfatal MI was based on Monitoring of Trends and Determinants in Cardiovascular Disease (MONICA) criteria [20]. We selected 3,869 controls among the participants who had survived to the end of the study period without having a MI, frequency-matched to cases with respect to the calendar year of recruitment, sex, and age (in 5-y bands). The National Bioethics Committee and the Data Protection Authority of Iceland approved the study protocol, and participants provided informed consent.

### Estimated Glomerular Filtration Rate

Creatinine measurements were carried out within days of the initial baseline examination (and, therefore, without knowledge of subsequent CHD status) using Jaffe methodology [21]. Values of eGFR were calculated using prediction equations involving circulating creatinine concentrations: either the four-variable Modification of Diet in Renal Disease (MDRD; the principal analysis in this paper; see below) or the Cockcroft-Gault (CG) equations (Table S1) [22,23]. Serum creatinine is included in each formula in units of mg/dl; eGFR_CG_ is expressed as ml/min, whereas eGFR_MDRD_ is expressed as ml/min/1.73 m^2^. In order to compare measurement of renal function between the two methods, eGFR_CG_ values were standardized for body surface area using the DuBois formula. Creatinine concentrations were also used alone as indicators of renal function. Other analytes were measured using standard methods, as described previously [24]. Blood urea nitrogen levels were not available.

### Statistical Analysis

Although creatinine measurements were made in all participants at baseline, nested case-control comparisons were used to enable analysis of data on biomarkers (e.g. inflammatory and haemostatic markers) that were available only in the nested case-control subset. Such comparisons were made by means of unmatched logistic regression fitted according to the unconditional maximum likelihood and progressively adjusted for possible confounding factors (Stata version 9, http://www.stata.com/). Principal analyses were prespecified to be by fifths of values of eGFR_MDRD_ in controls, with further subdivision of the lowest fifth into three groups (designated 5.1, 5.2, and 5.3). Subsidiary analyses were done using eGFR_CG_ and creatinine levels. To facilitate comparisons with previous studies of eGFR and cardiovascular outcomes secondary analyses were also performed using guideline classification of kidney function and on the basis of eGFR < 60 or ≥ 60 ml/min/1.73 m^2^ (eGFR <60 ml/min/1.73 m^2^ is considered the threshold for CKD) [25]. Odds ratios (ORs) were based on baseline eGFR. Analyses to investigate the shape of the eGFR-CHD association were conducted for groups of baseline eGFR values, with corresponding 95% confidence intervals (CIs) estimated from floated variances [26]. Subgroup analyses were prespecified by type of CHD outcome, age, sex, smoking habits, body mass index (BMI), systolic blood pressure, concentrations of serum lipids, blood glucose, haemoglobin, and uric acid. Linear regression was used to assess the association between eGFR and baseline characteristics among controls after adjustment for established cardiovascular risk factors (*p*-values for the *t*-statistic from regression of GFR on each characteristic are reported). To quantify within-person variability in levels of eGFR, that is, the extent to which an individual's eGFR measurements vary around a long-term average eGFR level, regression dilution ratios were estimated from a linear regression of the available repeat measurements [27,28] made in samples collected at an interval of about 12 y apart in 379 individuals, approximately the midpoint of this study's follow-up duration. ORs have not been corrected for regression dilution in the present study to allow direct comparison with previous work.

### Updated Meta-analysis

**Figure 1:**
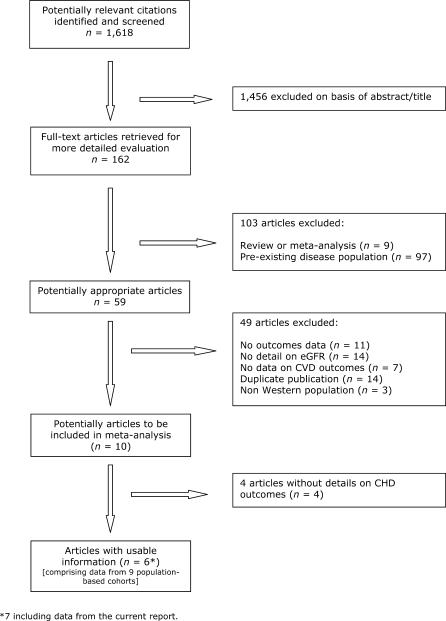
Summary of Meta-Analysis Flow

A meta-analysis of prospective studies published before March 2007 with greater than a year's follow-up and recruited from Western population–based sampling frameworks was performed (Figure 1). Studies were sought using computer-based databases (MEDLINE, EMBASE and Science Citation Index), by scanning the reference lists of articles identified for all relevant studies and review articles (including meta-analyses) and hand searching of relevant journals. The computer-based searches combined search terms related to renal function (e.g. “creatinine”, “glomerular filtration rate”, “kidney function”) and coronary disease (e.g. “myocardial infarction”, “atherosclerosis”, “coronary heart disease”, and “coronary stenosis”) without language restriction. Data were extracted independently by two investigators, using a prepiloted data extraction form (discrepancies were resolved by discussion and by adjudication of a third reviewer). One large study was not eligible for inclusion in this review because it involved a sample of insurance screenees identified at outpatient clinics, rather than a population-based sample [12]. The analysis was restricted to nonfatal MI or coronary death. Odds and hazard ratios were assumed to approximate the same measure of relative risk. To avoid potential biases, analyses involved only within-study comparisons (i.e., cases and controls were only directly compared within each cohort). We combined the results of the studies by using a random effects model. Heterogeneity was assessed by standard *χ* ^2^ tests and the *I* ^2^ statistic, which describes the percentage of variation in the log ORs that is attributable to genuine differences across studies rather than random error [29].

## Results

The mean age at CHD event among cases in the Reykjavik Study was about 66 y (standard deviation 8 y). As expected, there were highly statistically significant differences between cases and controls with respect to established cardiovascular risk factors (Table 1). Baseline creatinine concentrations and eGFR_MDRD_ showed no significant differences between cases and controls, whereas eGFR_CG_ was significantly higher in cases than in controls (a surprising finding consistent with the possibility of a nonlinear effect, as described below).

**Table 1:**
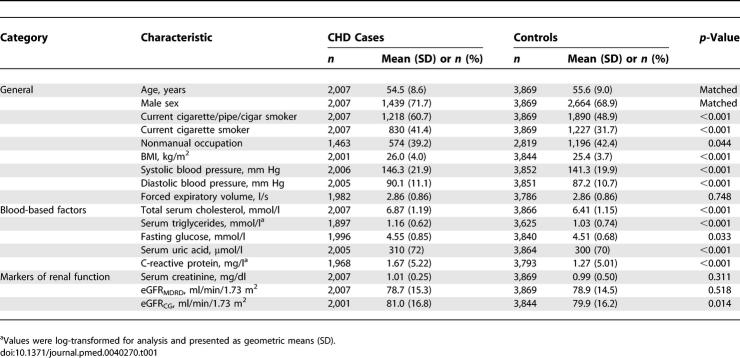
Baseline Characteristics of Patients Who Developed CHD during the Follow-up and Controls

### Baseline Associations and within-Person Variability

Values of eGFR were positively and highly significantly associated with male sex and smoking (*p* < 0.0001) and, somewhat less significantly, with manual occupation (*p* < 0.05). After adjustment for age, sex, period of recruitment, BMI, and smoking status there were highly significant inverse associations of eGFR with age, forced expiratory volume in one second, total cholesterol, triglycerides, haemoglobin, haematocrit, tissue plasminogen activator antigen and uric acid (*p* < 0.0001) and, somewhat less significantly, with diastolic blood pressure, albumin, and von Willebrand factor (*p* < 0.001) (Tables 2 and S2). In the participants who provided paired measurements at baseline and about 12 y later, the regression dilution ratios were as follows: serum creatinine 0.64 (95% CI 0.55–0.72), eGFR_MDRD_ 0.43 (95% CI 0.36–0.50), and eGFR_CG_ 0.51 (95% CI 0.45–0.58). These reproducibility values for markers of renal function were generally similar to the values noted in the same participants for total cholesterol 0.59 (0.51–0.67) and systolic blood pressure 0.65 (0.54–0.77).

**Table 2:**
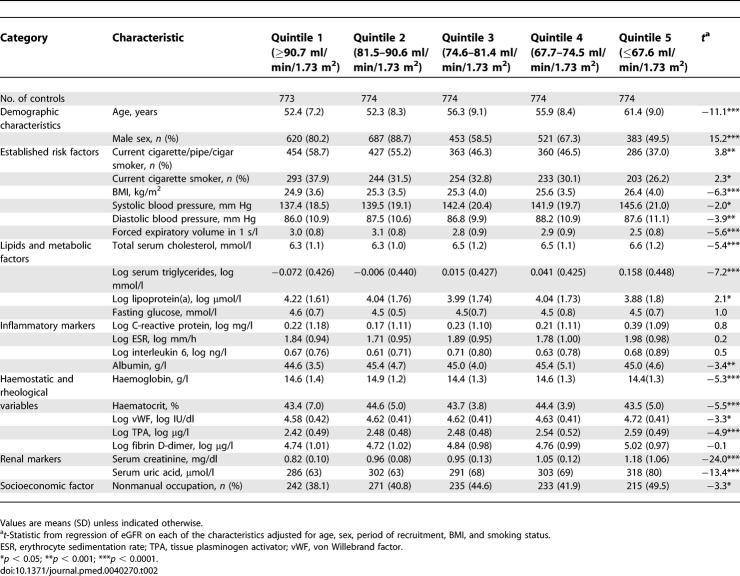
Baseline Correlates of eGFR_MDRD_ in Controls

### Renal Function and Incident Coronary Heart Disease

**Figure 2:**
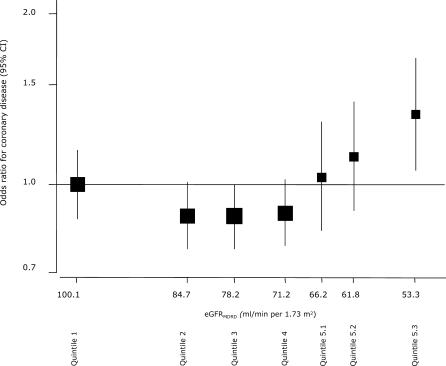
Association between Estimated Baseline GFR and CHD Adjusted for Age, Sex, Period, Smoking Status, and Other Established Risk Factors eGFR was calculated using Modification of Diet in Renal Disease equation. The size of the data markers is proportional to the inverse of the variance of the ORs. 95% CIs are calculated using floating-variance. Established risk factors included total cholesterol, triglycerides (log transformed), systolic blood pressure, and BMI. eGFR_MDRD_, overall *χ* ^2^ with 6 df = 14.6, *p* = 0.02.

Age-, sex-, and time period-adjusted analyses involving comparisons of fifths of eGFR yielded weak and nonsignificant ORs for CHD (Table 3), except in the lowest category of the lowest fifth of eGFR_MDRD_, which yielded an OR of 1.62 (95% CI 1.26–2.09), which fell to 1.33 (95% CI 1.01–1.75) after further adjustment for several established cardiovascular risk factors. Similar findings were observed for the same categories of low eGFR_CG_ and of serum creatinine concentration, giving adjusted ORs of 1.24 (95% CI 0.88–1.75) and 1.40 (95% CI 1.09–1.80), respectively (Table S3). Additional adjustment for C-reactive protein made little difference to the adjusted ORs (see Table 3 and Table S3 legends). Figures 2 and S1 plot the adjusted ORs for CHD by fifths of each of eGFR_MDRD_ and of eGFR_CG_, suggesting the possibility of a nonlinear effect in individuals with the lowest category of renal function (*p* = 0.02 and *p* = 0.002, respectively). Subsidiary analyses using the cut point of <60 versus ≥60 ml/min/1.73 m^2^ in eGFR yielded similar findings (Table S4). There was no substantial variation in the strength of association between eGFR (comparing individuals below 60 ml/min/1.73 m^2^ with those with higher values) and CHD risk at different levels of established cardiovascular risk factors; in particular, there were no significant interactions (*χ* ^2^ test for interaction *p* > 0.05) with sex, smoking status, BMI, total cholesterol, serum triglycerides, systolic blood pressure, blood glucose levels, and haematocrit (either with eGFR_MDRD_ or eGFR_CG_, Figure S2). The OR may have been somewhat higher for nonfatal MI (OR 1.75, 95% CI 1.29–2.39) than for fatal CHD (OR 1.19, 95% CI 0.90–1.56) (*p*-value for difference = 0.06). The ORs were largely unchanged when analyses excluded CHD cases recorded in the first 5 y of follow-up.

**Table 3:**
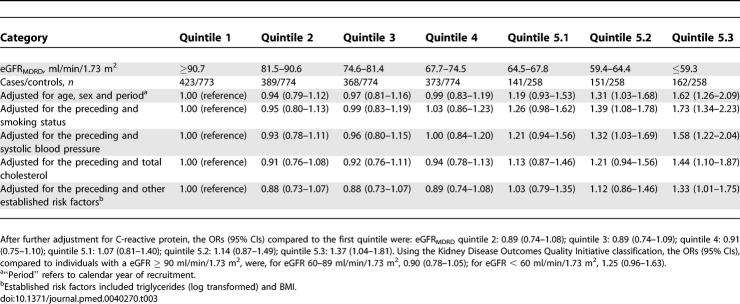
Relative Odds (95% CI) of CHD According to eGFR_MDRD_

### Updated Meta-analysis

**Figure 3:**
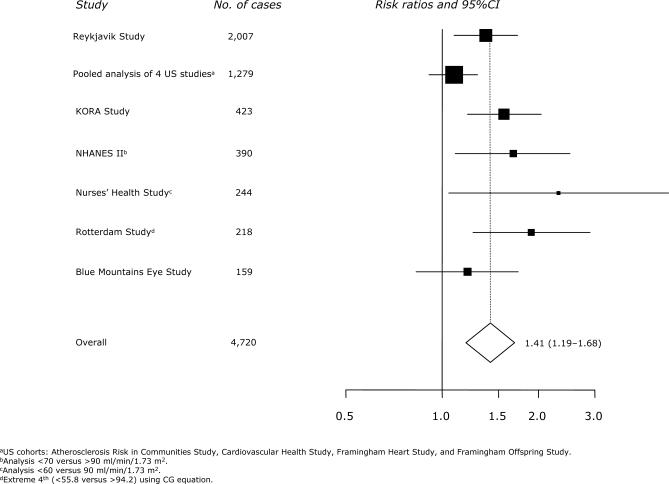
Meta-analysis of Reported Data from Prospective Studies in Essentially General Western Populations of CHD Risk in Individuals with eGFR_MDRD_ of <60 versus ≥ 60 ml/min/1.73 m^2^, with Adjustments Reported for Several Established Cardiovascular Risk Factors The size of the data markers is proportional to the inverse of the variance of the risk ratios. Overall estimate calculated using fixed effect meta-analysis. Random effects overall relative risk 1.32, 95% CI 1.19–1.47. Test for heterogeneity: *χ* ^2^ = 12.9 with 6 df, *p* = 0.045; *I* ^2^ = 56% (95% CI 0%–80%). For adjustments see Table 3. KORA, The MONICA/KORA Augsberg Study; NHANES, National Health and Nutrition Examination Survey.

We identified ten relevant prospective studies (identified in six previous reports, plus the current study) on eGFR and cardiovascular disease in essentially general populations (i.e., cohorts not selected on the basis of preexisting disease) that could be included in the present analysis because they reported specifically on CHD outcomes (Table 4) [11,13–17]. The studies were conducted in Western Europe [14,15], the US [11,16,17], and Australia [13]. All but one [17] involved both men and women, all involved predominantly middle-aged participants, all used standard creatinine assay methods (typically the Jaffe method), all but one [14] employed eGFR_MDRD_, and all adjusted risk estimates for several established cardiovascular risk factors. Reported mean levels (and distributions) of eGFR among these studies were similar (Table 4). There was no clear evidence of heterogeneity among the findings of the seven available published reports (*χ* ^2^ = 12.9 with 6 df, *p* = 0.045; *I* ^2^ = 56%, 95% CI 0%–80%), nor was there any evidence of selective publication. Using only within-study comparisons, a combined random effects meta-analysis of these reports (including the present study), involving a total of 4,720 incident CHD cases, yielded an adjusted risk ratio of 1.41 (95% CI 1.19–1.68) in individuals with baseline eGFR below versus those above 60 ml/min/1.73m^2^. A fixed-effect meta-analysis gave a relative risk of 1.32 (95% CI 1.19–1.47, Figure 3).

**Table 4:**
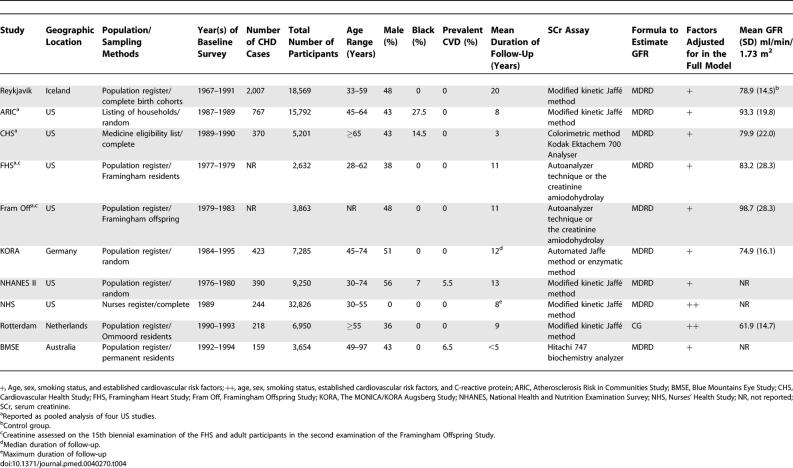
Characteristics of Prospective Studies of eGFR and CHD in Essentially General Western Populations

## Discussion

In the largest single population-based study of eGFR and CHD to our knowledge thus far, we have shown that there are no strong associations between lower-than-average eGFR and CHD risk in apparently healthy adults over most of the range in renal function. A moderate association may, however, exist among individuals with very low eGFR, i.e., those with eGFR near or less than 60 ml/min/1.73 m^2^ of body surface area. This cut point coincides with the definition of CKD suggested by the American National Kidney Foundation [25]. Such an association implies a nonlinear effect of low eGFR on CHD risk, a possibility that requires evaluation in larger numbers. Moderate associations among individuals with eGFR near or less than 60 ml/min/1.73 m^2^ of body surface area were consistently observed with CHD risk in many clinically relevant subgroups, such as in women and men and at different levels of established risk factors and other characteristics (e.g., blood glucose levels and haematocrit). Our updated meta-analysis reinforces the validity and generalisability of the new data from the Reykjavik study; examination of several study characteristics in our review did not identify important sources of heterogeneity. Finally, our data demonstrate that the decade-to-decade consistency in renal function in apparently healthy adults is similar to that for blood pressure or total blood cholesterol, although larger assessments will be required to assess whether this variability differs importantly at different levels of renal function.

These findings from the Reykjavik study may have several implications. First, the apparent lack of any association of renal function in the normal range with CHD risk implies that strategies which aim to shift the population distribution of renal function may not be optimum for the purposes of CHD prevention [30]. By contrast, suggestions of associations of lower-than-normal eGFR with CHD risk encourage further investigation of targeting existing cardioprotective interventions (such as lipid- and blood pressure-lowering agents [6,31–33]) in the 5%–10% of the general western adult population who fulfil the criteria for CKD [34]. Second, highly significant associations of low eGFR with tissue plasminogen activator antigen that have been observed stimulate interest in the potential relevance of haemostasis to renal function [35], whereas there were no strong cross-sectional associations of low eGFR with C-reactive protein, interleukin 6, and lipoprotein(a) [36,37]. Third, our data highlight the need for studies that can determine whether eGFR is primarily a risk factor for CHD or mainly a marker of subclinical cardiovascular diseases (i.e., reflecting the extent and severity of atherosclerosis in renal and other vascular beds such as the coronary circulation [10,38]). The former possibility has been highlighted in previous reports of significant correlations between coronary and renal arteriosclerosis in the absence of any overt clinical CKD or CHD [39,40]. The latter possibility is suggested by the modest residual association observed between low eGFR and CHD risk after adjustment for measured levels of established risk factors, as adjustment for the full impact of these risk factors might have reduced the association still further (or even abolished it).

The strengths and potential limitations of the present study merit careful consideration. Our report involves new data on eGFR levels and incident CHD that almost double the available evidence on major CHD outcomes in population-based studies and avoids the potentially misleading analysis of heterogeneous cardiovascular disease outcomes used in some studies. We identified participants in population registers, had high response and follow-up rates, used robust methods to ascertain CHD outcomes, and minimized potential biases by exclusion of participants with prevalent diabetes, CHD, and stroke. Concomitant measurements of several established risk factors and emerging markers in the same participants enabled adjustment for a range of possible confounding factors. Although our assays produced creatinine values comparable with those in earlier studies (Table 4), eGFR might not be optimal for use in general populations, because standard prediction equations used to calculate it have been developed in patients with kidney disease [21,41,42]. Furthermore, serum creatinine is, of course, an imperfect indicator of GFR because it is influenced by creatinine generation and tubular secretion, but the eGFR equations employed in the current analyses are in widespread use and take into account age, sex, and weight (the latter only in the Cockcroft-Gault equation). Future studies may improve on eGFR methods by recalibration of serum creatinine assays to the isotope-dilution mass spectrometry standard [43,44] and by measurement of potentially more sensitive markers such as serum cystatin C [45] or symmetric and asymmetric dimethylarginine (SDMA and ADMA) [46, 47]. The current analyses do not address low eGFR and CHD risk among ethnic groups who may be particularly susceptible to the effects of impaired renal function [48].

### Conclusion

Our new large-scale data in apparently healthy Western adults—reinforced by a meta-analysis of previous relevant studies in similar populations (i.e., Western European, North American, or Australian)—suggest that levels of eGFR compatible with the definition of CKD are associated with about a 40% increased risk in subsequent CHD. These findings could have implications for further understanding the pathogenesis of CHD and targeting existing cardioprotective interventions.

## Supporting Information

Figure S1 Association between Baseline eGFR_CG_ and CHD Adjusted for Age, Sex, Period, Smoking Status and Other Established Risk Factors (32 KB PPT)

Figure S2 Risk of CHD in Individuals with Baseline eGFR_MDRD_ and eGFR_CG_ < 60 Versus ≥ 60 ml/min/1.73 m^2^, Grouped According to Various Characteristics (74 KB PPT)

Table S1 Characteristics of the CG Equation, the Simplified MDRD Equation, and Creatinine to Estimate the Glomerular Filtration Rate (34 KB DOC)

Table S2 Baseline Correlates of eGFR_CG_ in Controls (90 KB DOC)

Table S3 Relative Odds (95% CI) of CHD According to the Baseline eGFR_CG_ and Creatinine Levels (57 KB DOC)

Table S4 Relative Odds (95% CI) of CHD According to the Baseline eGFR_MDRD_ and eGFR_CG_ <60 Versus ≥ 60 ml/min/1.73 m^2^
(38 KB DOC)

## Abbreviations

BMI: body mass index
CG: Cockcroft-Gault
CHD: coronary heart disease (defined as nonfatal myocardial infarction or coronary death)
CI: confidence interval
CKD: chronic kidney disease
eGFR: estimated glomerular filtration rate
MDRD: Modification of Diet in Renal Disease
MI: myocardial infarction
OR: odds ratio
