# Supplementary material for: Renal Function and Risk of Coronary Heart Disease in General Populations: New Prospective Study and Systematic Review
Source: PLoS Med. 2007 Sep 4;4(9):e270. doi: 10.1371/journal.pmed.0040270 (PMC1961630; doi:10.1371/journal.pmed.0040270)
Supplement: Supplementary file 1 [file pmed.0040270.st001.doc]

**Supplementary table 1.** Characteristics of the CG Equation, the Simplified MDRD Equation, and Creatinine to Estimate the Glomerular Filtration Rate

|  | ***Method to estimate Glomerular Filtration Rate*** | | |
| --- | --- | --- | --- |
| *Prediction Equation* | **Cockcroft-Gault** | **MDRD “4-variables”** | **Creatinine** |
| *Formula* | [(140 – age) x weight/(72 x SCr)] x (0.85 if female) | 186.3 x SCr^-1.154^ x Age^-0.203^ x (0.742 if female) x (1.21 if black) | N/A |
| *“Gold standard” used in calibration of formula* | 24-h urinary creatinine collections | ^125^I-iothalamate renal clearance | N/A |
| *Possible advantages* | More accurate than MDRD in people with normal glomerular function and in people aged <65 years | More precise and more accurate than the CG equation in CKD patients and people aged >65 years | Easy to use measure |
| *Possible disadvantages* | Validated using creatinine clearance as gold standard  Overestimation of GFR  Inferior to MDRD in CKD patients | Developed in CKD populations only  Underestimation of GFR in healthy populations | Dependent on muscle mass, age, sex.  Rise in serum level only when GFR is decreased by 60% of its normal |

Age is included in years, weight in kg and serum creatinine (SCr) in mg/dl

CKD, chronic kidney disease; GFR, glomerular filatration rate; MDRD, Modification of Diet in Renal Disease; CG, Cockcroft-Gault; N/A, not available
