# Supplementary material for: Renal Function and Risk of Coronary Heart Disease in General Populations: New Prospective Study and Systematic Review
Source: PLoS Med. 2007 Sep 4;4(9):e270. doi: 10.1371/journal.pmed.0040270 (PMC1961630; doi:10.1371/journal.pmed.0040270)
Supplement: Supplementary file 2 [file pmed.0040270.st002.doc]

**Supplementary table 2.** Baseline Correlates of eGFR_CG_ in Controls

|  | ***Estimated eGFR_CG_ ( mL/min/1.73 m^2^)*** | | | | |  |
| --- | --- | --- | --- | --- | --- | --- |
|  | **Quintile 1** | **Quintile 2** | **Quintile 3** | **Quintile 4** | **Quintile 5** |  |
|  | ≥ 92.3 | 82.7-92.2 | 75.6-82.6 | 67.0-75.5 | ≤ 66.9 | t value^a^ |
| **No. of controls** | 768 | 769 | 769 | 769 | 769 |  |
| **Demographic characteristics** |  |  |  |  |  |  |
| Age, years | 49.2 (6.5) | 51.7 (6.3) | 54.4 (6.4) | 57.2 (7.4) | 65.2 (8.3) | -38.5*** |
| Male sex (%) | 591 (76.9) | 560 (72.8) | 564 (73.3) | 513 (66.7) | 424 (55.1) | 3.2* |
| **Established risk factors** |  |  |  |  |  |  |
| Current cigarette/pipe/cigar smoker (%) | 442 (57.6) | 378 (49.2) | 381 (49.5) | 375 (48.8) | 302 (48.9) | 3.5** |
| Current cigarette smoker(%) | 276 (35.9) | 245 (31.9) | 244 (31.7) | 233 (46.0) | 220 (45.2) | 2 |
| Body mass index, kg/m^2^ | 27.2 (4.0) | 25.7 (3.5) | 25.5 (3.4) | 24.6 (3.3) | 24.0 (3.6) | 29.9*** |
| Systolic blood pressure, mmHg | 138.9 (19.0) | 140.8 (19.4) | 141.1 (19.1) | 141.3 (20.8) | 144.7 (20.8) | -1.6 |
| Diastolic blood pressure, mmHg | 87.5 (11.2) | 88.3 (10.8) | 87.7(10.3) | 86.8 (10.6) | 85.8 (10.5) | -3.5** |
| Forced expiratory volume in 1 s/L | 3.2 (0.8 ) | 3.0 (0.8 ) | 2.9 (0.8 ) | 2.8 (0.8) | 2.4 (0.8) | -3.4** |
| **Lipids and metabolic factors** |  |  |  |  |  |  |
| Total serum cholesterol, mmol/L | 6.3 (1.2) | 6.4(1.1) | 6.5 (1.1) | 6.5 (1.1) | 6.5 (1.2) | -5.4*** |
| Log serum triglycerides, log mmol/L | 0.037 (0.455) | -0.001 (0.456) | 0.039 (0.438) | 0.003 (0.432) | 0.063 (0.409) | -6.8*** |
| Log lipoprotein(a), log μmol/L | 4.14 (1.64) | 4.04 (1.74) | 4.02 (1.78) | 3.99 (1.76) | 3.97 (1.75) | 2.2* |
| Fasting glucose, mmol/L | 4.6 (0.7) | 4.5 (0.7) | 4.5 (0.6) | 4.5 (0.9) | 4.5(0.6) | 1.0 |
| **Inflammatory markers** |  |  |  |  |  |  |
| Log C-reactive protein, log mg/L | 0.29 (1.15) | 0.21 (1.09) | 0.17 (1.12) | 0.18 (1.11) | 0.37 (1.12) | 0.7 |
| Log erythrocyte sedimentation rate, log mm/hr | 1.84 (0.93) | 1.84 (0.96) | 1.74 (0.96) | 1.82 (0.99) | 1.95 (0.99) | 0.1 |
| Log interleukin 6, log ng/L | 0.65 (0.74) | 0.62 (0.73) | 0.66 (0.76) | 0.65 (0.87) | 0.75(0.85) | 0.4 |
| Albumin, g/L | 44.8 (3.2) | 45.3 (4.4) | 45.5 (4.9) | 45.2 (4.6) | 44.6 (4.8) | -3.4** |
| **Hemostatic and rheological variables** |  |  |  |  |  |  |
| Haemoglobin, g/L | 14.7 (1.4) | 14.7 (1.3) | 14.7 (1.2) | 14.5 (1.2) | 14.2 (1.3) | -5.4*** |
| Haematocrit (%) | 43.7 (6.9) | 44.3 (4.6) | 44.4 (4.7) | 44.1 (4.2) | 43.2 (4.6) | -5.8*** |
| Log von Willebrand factor, log IU/dL § | 4.58 (0.42) | 4.57 (0.42) | 4.62 (0.41) | 4.64 (0.41) | 4.76 (0.39) | -3.5** |
| Log tissue plasminogen activator, log μg/L § | 2.51 (0.48) | 2.49 (0.51) | 2.53 (0.47) | 2.46 (0.52) | 2.52 (0.49) | -4.7*** |
| Log fibrin D-dimer, log μg/L § | 4.58 (1.03) | 4.66 (1.00) | 4.75 (0.97) | 4.89 (0.96) | 5.17 (0.95) | -0.4 |
| **Renal markers** |  |  |  |  |  |  |
| Serum creatinine, mg/dL | 0.85 (0.12) | 0.94 (0.12) | 1.0 (0.13) | 1.03 (0.14) | 1.14 (1.06) | -25.0*** |
| Serum uric acid, µmol/L | 298 (66) | 300 (70) | 302 (65) | 298 (66) | 303 (79) | -13.0*** |
| **Socioeconomic factor** |  |  |  |  |  |  |
| Non-manual occupation, n (%) | 119 (28.3) | 134 (30.0) | 132 (45.7) | 138 (34.5) | 105 (28.0) | -2.4* |

^a^ t statistic from regression of eGFR_CG_ on each of the characteristics adjusted for age, sex, period of recruitment, BMI and smoking status. Values are means (SD) unless indicated otherwise. *p<0.05; **p<0.001; ***p<0.0001
