# Supplementary material for: Renal Function and Risk of Coronary Heart Disease in General Populations: New Prospective Study and Systematic Review
Source: PLoS Med. 2007 Sep 4;4(9):e270. doi: 10.1371/journal.pmed.0040270 (PMC1961630; doi:10.1371/journal.pmed.0040270)
Supplement: Supplementary file 3 [file pmed.0040270.st003.doc]

**Supplementary table 3.** Relative Odds (95% CI) of CHD According to the Baseline eGFR_CG_ and Creatinine Levels

|  | **Quintile 1** | **Quintile 2** | **Quintile 3** | **Quintile 4** | **Quintile 5.1** | **Quintile 5.2** | **Quintile 5.3** |
| --- | --- | --- | --- | --- | --- | --- | --- |
| ***eGFR_CG_  ( mL/min/1.73 m^2^)*** | ≥ 92.3 | 82.7-92.2 | 75.6-82.6 | 67.0-75.5 | 63.0-66.9 | 56.2-63.0 | ≤ 56.1 |
| ***No. of cases/controls*** | 471/768 | 372/769 | 388/769 | 367/769 | 140/256 | 149/256 | 114/257 |
| Adjusted for age, sex and period^a^ | 1.00 (reference) | 0.83 (0.70-0.99) | 0.90 (0.75-1.07) | 0.89 (0.74-1.07) | 1.09 (0.84-1.41) | 1.27 (0.97-1.66) | 1.11 (0.83-1.50) |
| Adjusted for the preceding and smoking status | 1.00 (reference) | 0.84 (0.71-1.00) | 0.90 (0.75-1.08) | 0.88 (0.73-1.06) | 1.09 (0.84-1.42) | 1.26 (0.96-1.66) | 1.09 (0.81-1.48) |
| Adjusted for the preceding and systolic blood pressure | 1.00 (reference) | 0.85 (0.72-1.02) | 0.94 (0.79-1.13) | 0.94 (0.78-1.13) | 1.18 (0.91-1.54) | 1.39 (1.05-1.82) | 1.14 (0.84-1.55) |
| Adjusted for the preceding and total cholesterol | 1.00 (reference) | 0.82 (0.69-0.98) | 0.88 (0.73-1.06) | 0.87 (0.72-1.06) | 1.15 (0.88-1.50) | 1.28 (0.97-1.69) | 1.04 (0.76-1.42) |
| Adjusted for the preceding and other established risk factors^b^ | 1.00 (reference) | 0.84 (0.69-1.01) | 0.94 (0.77-1.15) | 0.99 (0.80-1.22) | 1.30 (0.97-1.73) | 1.47 (1.08-1.99) | 1.24 (0.88-1.75) |
| ***Serum creatinine^c^, mg/dL*** |  |  |  |  |  |  |  |
| ***No. of cases/controls*** | 416/774 | 347/774 | 378/774 | 397/774 | 165/258 | 136/258 | 168/257 |
| Adjusted for age, sex and period^a^ | 1.00 (reference) | 1.01 (0.85-1.20) | 0.99 (0.83-1.18) | 1.08 (0.91-1.29) | 1.32 (1.04-1.67) | 1.05 (0.82-1.35) | 1.64 (1.30-2.06) |
| Adjusted for the preceding and smoking status | 1.00 (reference) | 1.01 (0.85-1.21) | 1.01 (0.84-1.20) | 1.12 (0.94-1.33) | 1.35 (1.06-1.71) | 1.10 (0.85-1.42) | 1.72 (1.36-2.17) |
| Adjusted for the preceding and systolic blood pressure | 1.00 (reference) | 0.99 (0.83-1.19) | 0.99 (0.83-1.18) | 1.09 (0.91-1.31) | 1.33 (1.05-1.69) | 1.07 (0.83-1.38) | 1.58 (1.25-2.00) |
| Adjusted for the preceding and total cholesterol | 1.00 (reference) | 1.00 (0.84-1.20) | 0.95 (0.79-1.14) | 1.05 (0.88-1.26) | 1.23 (0.96-1.56) | 1.01 (0.78-1.31) | 1.47 (1.16-1.88) |
| Adjusted for the preceding and other established risk factors^b^ | 1.00 (reference) | 0.99 (0.82-1.20) | 0.91 (0.76-1.11) | 1.02 (0.84-1.23) | 1.17 (0.91-1.51) | 0.93 (0.71-1.21) | 1.40 (1.09-1.80) |

^a^ “Period” refers to calendar year of recruitment.

^b^ Established risk factors included triglycerides (log transformed) and BMI

^c^ Sex-specific serum creatinine quintiles (mg/dL): males (quintile 1, ≤ 0.92; quintile 2, 0.93-0.99; quintile 3, 1.00-1.06; quintile 4, 1.07-1.15; quintile 5.1, 1.16-1.20; quintile 5.2, 1.21-1.26; quintile 5.3, ≥1.27); females (quintile 1, ≤ 0.76; quintile 2, 0.77-0.83; quintile 3, 0.83-0.89; quintile 4, 0.90-0.97; quintile 5.1, 0.98-1.02; quintile 5.2, 1.03-1.08; quintile 5.3, ≥1.09)

After further adjustment for C-reactive protein, the odds ratios (95% CI) compared to the first quintile were: *eGFR_CG_* quintile 2, 0.85 (0.70-1.03); quintile 3, 0.96 (0.78-1.17); quintile 4, 1.01 (0.82-1.26); quintile 5.1, 1.33 (0.99-1.78); quintile 5.2, 1.52 (1.12-2.06); quintile 5.3, 1.32 (0.93-1.87); serum creatinine quintile 2, 0.99 (0.82-1.20); quintile 3, 0.92 (0.76-1.11); quintile 4, 1.03 (0.85-1.25); quintile 5.1, 1.20 (0.93-1.55); quintile 5.2, 0.92 (0.70-1.21); quintile 5.3, 1.42 (1.10-1.83).
