# Supplementary material for: Renal Function and Risk of Coronary Heart Disease in General Populations: New Prospective Study and Systematic Review
Source: PLoS Med. 2007 Sep 4;4(9):e270. doi: 10.1371/journal.pmed.0040270 (PMC1961630; doi:10.1371/journal.pmed.0040270)
Supplement: Supplementary file 4 [file pmed.0040270.st004.doc]

**Supplementary table 4.** Relative Odds (95% CI) of CHD According to the Baseline eGFR_MDRD_ and eGFR_CG_ <60 Versus ≥ 60 ml/min/1.73 m^2^

|  | **Odds ratio (95% CI)** |
| --- | --- |
| ***eGFR_MDRD_ (*** ***mL/min/1.73 m^2^)***  ***(GFR <60: cases 191, controls 283; GFR ≥ 60: cases 1886, controls 3648)*** | ***GFR <60 vs ≥ 60*** |
| Adjusted for age, sex and period^a^ | 1.50 (1.21, 1.85) |
| Adjusted for the preceding and smoking status | 1.55 (1.25, 1.92) |
| Adjusted for the preceding and systolic blood pressure | 1.47 (1.19, 1.82) |
| Adjusted for the preceding and total cholesterol | 1.40 (1.12, 1.74) |
| Adjusted for the preceding and other established risk factors^b^ | 1.37 (1.09, 1.72) |
| ***eGFR_CG_  ( mL/min/1.73 m^2^)***  ***(GFR <60: cases 196, controls 368; GFR ≥ 60: cases 1875, controls 3537)*** |  |
| Adjusted for age, sex and period^a^ | 1.34 (1.08, 1.66) |
| Adjusted for the preceding and smoking status | 1.31 (1.06, 1.62) |
| Adjusted for the preceding and systolic blood pressure | 1.32 (1.07, 1.64) |
| Adjusted for the preceding and total cholesterol | 1.30 (1.05, 1.62) |
| Adjusted for the preceding and other established risk factors^b^ | 1.40 (1.11, 1.75) |

eGFR, estimated glomerular filatration rate; MDRD, Modification of Diet in Renal Disease; CG, Cockcroft-Gault.

^a^ “Period” refers to calendar year of recruitment.

^b^ Established risk factors included total cholesterol, triglycerides (log transformed), systolic blood pressure and BMI.
